# Central and Peripheral Mechanisms of Low Exercise Capacity in Prematurely Born Adults

**DOI:** 10.1002/cph4.70049

**Published:** 2025-09-12

**Authors:** Giorgio Manferdelli, Gregory P. Barton, Tony G. Babb, Grégoire P. Millet, Michael D. Nelson, Benjamin D. Levine, Kara N. Goss

**Affiliations:** ^1^ Institute for Exercise and Environmental Medicine Texas Health Presbyterian Hospital Dallas Dallas Texas USA; ^2^ Department of Internal Medicine The University of Texas Southwestern Medical Center Dallas Texas USA; ^3^ Institute of Sport Sciences University of Lausanne Lausanne Switzerland; ^4^ Department of Kinesiology The University of Texas at Arlington Arlington Texas USA; ^5^ Department of Pediatrics The University of Texas Southwestern Medical Center Dallas Texas USA; ^6^ Parkland Health Dallas Texas USA

**Keywords:** adults, exercise, premature birth, skeletal muscle, stroke volume

## Abstract

**Purpose:**

Premature birth (< 37 weeks gestation) is associated with lower exercise capacity. However, the specific underlying mechanisms remain poorly defined. This study investigated the mechanisms of exercise limitation across the oxygen transport chain in preterm‐born adults with normal resting cardiopulmonary function but exertional dyspnea.

**Methods:**

10 preterm born (6F, age: 30 ± 5 years, body mass index [BMI]: 27.0 ± 6.3 kg/m^2^, gestational age: 30 ± 3 weeks) and 8 term born (3F, age: 29 ± 5 years, BMI: 25.4 ± 4.6 kg/m^2^, gestational age: 40 ± 0 weeks) adults performed resting spirometry and a cardiopulmonary exercise test, consisting of two 5‐min submaximal cycling exercises (30 and 60 W), followed by an incremental protocol to exhaustion. We measured breath‐by‐breath gas exchange (custom designed system), heart rate (HR, 12‐lead ECG), cardiac output (Q̇c, acetylene rebreathe), and calculated arterial–venous oxygen difference (a‐*v*O_2_diff, Fick equation).

**Results:**

Oxygen uptake (V̇O_2_) was similar between groups at rest, 30 and 60 W. At peak, compared to term‐born peers, preterm adults showed lower power output (108 ± 18 vs. 208 ± 69 W, *p* < 0.001), V̇O_2_ (1.58 ± 0.29 vs. 2.52 ± 0.85 L/min, *p* = 0.017), Q̇c_index_ (7.5 ± 1.0 vs. 8.9 ± 1.6 L/min/m^2^, *p* = 0.057), while a‐*v*O_2_diff (12.6 ± 1.7 vs. 14.1 ± 1.6 mL/dL, *p* = 0.096) and HR were similar between groups (175 ± 16 vs. 185 ± 8 bpm, *p* = 0.104). The increase in stroke volume index from rest to peak exercise was blunted in preterm compared to term‐born adults (8 ± 7 vs. 15 ± 6 mL/m^2^, *p* = 0.032).

**Conclusion:**

Preterm born adults present with lower exercise capacity compared to age‐matched peers born at term. Central mechanisms, primarily stroke volume, underlie exercise limitation in this population.

## Graphical abstract

The lower exercise capacity described in preterm born adults is associated with impaired stroke volume augmentation in response to incremental exercise intensity, thus to a lower cardiac output response to exercise, compared to age‐matched individuals born at term.

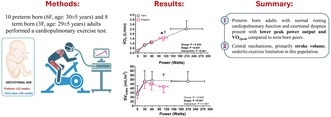

## 1 Introduction

Premature birth, or < 37 weeks' gestation, is increasingly common, with over 13 million premature newborns born every year (Liang et al. 2024). While premature birth and its complications are among the leading causes of mortality among children under 5 years of age, 95% of children born preterm survive into adulthood. Nevertheless, preterm‐born individuals are at greater risk for respiratory, cardiac, and vascular diseases later in life (Ahmed et al. 2024; Raju et al. 2017).

Recent studies in preterm survivors have shown persistent abnormalities of respiratory and cardiovascular function during adulthood (Goss et al. 2018, 2019, 2020; Haraldsdottir et al. 2020; Macdonald et al. 2021). As these systems must work synergistically to elicit an appropriate response to exercise, it is not surprising that exercise capacity and cardiorespiratory fitness (i.e., peak oxygen uptake [V̇O_2peak_]) have been almost universally demonstrated to be reduced in this population compared to age‐matched individuals born at term (Haraldsdottir et al. 2020; Vrijlandt et al. 2006; Lovering et al. 1985; Duke et al. 2014; Farrell et al. 2015; Manferdelli, Narang, Bourdillon, et al. 2023; Haraldsdottir et al. 2018). Importantly, adults born very preterm have a lower exercise capacity than term‐born peers despite having similar physical activity levels (Tikanmaki et al. 2017). However, due to the continuing improvement of neonatal care treatments and likely the heterogeneity of this population (i.e., lifestyle, socioeconomic status, presence of bronchopulmonary dysplasia), the sites of functional limitation to exercise capacity in adult survivors of premature birth remain debated.

Following premature birth, variable degrees of airflow obstruction, alveolar and pulmonary vascular density, and overall pulmonary limitation to exercise have been consistently demonstrated (reviewed by Duke et al. (2022)). However, in the absence of bronchopulmonary dysplasia with persistent resting airflow obstruction, pulmonary physiology does not explain the reduced aerobic exercise capacity in preterm adults (Vollsaeter et al. 2015; Huckstep et al. 2021), but rather the functional and structural cardiac impairments previously described in preterm adults seem to play a more critical role (Telles et al. 2020; Schuermans and Lewandowski 2022). Indeed, adults born preterm present with smaller biventricular chamber size, lower biventricular mass, and reduced left ventricular (LV) longitudinal systolic, diastolic, and rotational function, despite similar LV ejection fraction (LVEF), compared to term‐born peers (Goss et al. 2020; Barton et al. 2024; Harris et al. 2021). The addition of a physiological stressor, like exercise, further accentuates these functional and structural deficits of the premature heart. The impaired LV response to exercise in preterm‐born adults is characterized by lower ejection fraction and cardiac output (Q̇c) during moderate‐to‐severe exercise intensity (Haraldsdottir et al. 2018, 2020; Manferdelli, Narang, Bourdillon, et al. 2023; Huckstep et al. 2018, 2021). Importantly, the heart rate response to exercise in adults born prematurely is comparable to term‐born peers, with reduced stroke volume reserve being the primary limitation to increase Q̇c during exercise (Haraldsdottir et al. 2020; Manferdelli, Narang, Bourdillon, et al. 2023). However, most of this evidence comes from studies in which the cardiac response to exercise was assessed imprecisely using thoracic bioimpedance, and therefore further investigations using more accurate and precise methods to measure stroke volume and Q̇c during exercise in this cohort are required.

The primary aim of this study was to investigate the cardiac mechanisms of exercise limitation in prematurely born but otherwise healthy adults. We hypothesized that impaired stroke volume reserve during heavy exercise underlies the blunted cardiac output response to exercise in preterm adults. Moreover, we sought to elucidate whether skeletal muscle may contribute to reduced exercise capacity in adults born prematurely. We hypothesized similar skeletal muscle oxygen extraction in preterm and term‐born adults.

## 2 Methods

### 2.1 Participants and Study Design

Eighteen healthy male and female adults volunteered and gave written informed consent to participate in this study. Eight participants were born at term and 10 were preterm. Preterm‐born participants (inclusion criteria: gestational age ≤ 32 weeks or birth weight < 1500 g) who previously participated in a cardiopulmonary screening study (Barton et al. 2024; Blair et al. 2025) at UT Southwestern Medical Center and had *normal* resting cardiac and lung function but exertional dyspnea were invited to return for an exercise study. Birth history was obtained from the Parkland Neonatal Intensive Care Unit Database or external neonatal records. The inclusion criteria for the term‐born participants were gestational age ≥ 37 weeks and birth weight ≥ 2500 g. Exclusion criteria for all participants comprised forced vital capacity (FVC) and forced expiratory volume in 1s (FEV_1_) < 70% of predicted value (using NHANES III equations), LVEF < 50% (measured by echocardiography), history of asthma, and inability to complete study procedures. Two preterm participants had a neonatal history of bronchopulmonary dysplasia. Each participant was studied at a single visit, during which they underwent a complete pulmonary function evaluation, followed by an incremental cardiopulmonary exercise test to exhaustion. Before study visits, all participants were instructed to refrain from vigorous exercise for 24 h and from alcohol and caffeine consumption in the 12 h leading up to the laboratory visit. Self‐reported physical activity was assessed using the Global Physical Activity Questionnaire (GPAQ). This study was performed according to the Declaration of Helsinki. Ethical approvals were obtained from the UT Southwestern Medical Center Institutional Review Board and all participants provided written informed consent.

### 2.2 Resting Pulmonary Function

Participants completed standard spirometry and lung volume assessment (model 6200 body plethysmograph, SensorMedics, Yorba Linda, CA) according to American Thoracic Society guidelines (Graham et al. 2019) in a temperature‐controlled laboratory (~22°C) at the Institute for Exercise and Environmental Medicine (IEEM) in Dallas, TX. Lung function parameters were expressed both in absolute terms and as a percentage of predicted values using established equations (Hankinson et al. 1999).

### 2.3 Cardiopulmonary Exercise Testing

Following 3 min of seated rest, participants completed two 5‐min submaximal exercise bouts on an electronically braked cycle ergometer (Lode Excalibur, Lode B.V., Groningen, The Netherlands), the first at 30 W and the latter at 60 W. After a 15‐min break, participants began to cycle at power output corresponding to moderate intensity exercise for 1 min; thereafter, power output was increased by 10–20 W every min until participants could no longer maintain the required cadence despite strong verbal encouragement. Throughout the tests, participants were instructed to maintain a pedaling frequency of 60 rpm. A customized breath‐by‐breath measurement system (Beck Integrative Physiological System, BIPS; KCBeck, Physiological Consulting, Liberty, UT) integrated with a mass spectrometer (Perkin‐Elmer 1100) was used to measure pulmonary gas exchange. Before each test, calibration of the analyzer was performed by using reference gases. Expiratory and inspiratory flows were measured at the mouth with a turbine flow device, which was calibrated before each test with the use of a 3‐L calibration syringe. Ventilatory mechanics parameters were collected using a custom dual pneumotach system (Spike 2 data acquisition and analysis package, Cambridge Electronic Design Limited, Cambridge, England). Heart rate (HR) was continuously monitored by a 12‐lead electrocardiogram (Xscribe 5 System and QStress System; Welch Allyn Inc., Skaneateles Falls, NY). Blood pressure was measured at rest, 30 W, 60 W, and at peak exercise by electrosphygmomanometry (Suntech Tango M2; Morrisville, North Carolina). Q̇c was determined by the acetylene rebreathe technique. This technique has been validated against direct Fick at rest and during both submaximal and maximal exercise (Hardin et al. 2020; Jarvis et al. 2007). Gas concentrations used for the acetylene rebreathe technique were: 9% helium, 0.6% acetylene, 45% oxygen, balance nitrogen. During resting measurements, participants were coached to maintain their respiratory rate at about 7–8 breaths in 20 s and tidal volume at about 1.5 L (measured with a turbine flow meter during patient‐determined inspiration). During exercise, participants typically met minimum requirements for respiratory rate and tidal volume (to ensure adequate gas mixing in the lung and a stable helium concentration) so minimal coaching was necessary.

Blood lactate concentration was measured at rest, during submaximal exercise, and within 2 min following exercise cessation. Borg ratings of perceived breathlessness and exertion (RPB, 0–10 scale and RPE, 6–20 scale, respectively) (Borg 1982) were collected at rest and during submaximal and maximal exercise. Maximal exhaustion was considered attained when at least three of the following parameters were observed: a respiratory exchange ratio > 1.10, a capillary lactate concentration ([La^−^]_b_) > 8 mmol·L^−1^ within 2 min after exercise cessation, a maximal heart rate (HR) > 85% of the participant's predicted maximal HR, or a RPE > 18. Stroke volume was calculated as the ratio between Q̇c and HR. The individual cardiac output response to exercise (∆Q̇c/∆V̇O_2_) was determined using a linear regression of the cardiac output over the oxygen uptake at rest, during the two submaximal workloads previously described, and at peak exercise. The mean Q̇c/V̇O_2_ slope for each group was obtained by averaging the individual regressed slopes. Total peripheral resistance (TPR) was calculated as the ratio of mean arterial pressure [DBP + 0.33 × (SBP‐DBP)] and Q̇c. Arterial–venous oxygen difference (a‐*v*O_2_diff) was calculated as the ratio between Q̇c and V̇O_2_ using the Fick principle. Q̇c and stroke volume were also indexed to body surface area (BSA) to allow comparison among individuals. Functional stroke volume reserve was calculated as the percent increase in stroke volume from rest to low‐intensity exercise equivalent to 30 W. The oxygen cost of cycling was calculated as the V̇O_2_/work rate slope from 30 W to peak exercise intensity. Ventilatory efficiency was assessed as the V̇_E_/V̇CO_2_ slope from rest to 30 W exercise.

## 3 Statistical Analysis

All data are presented as mean ± standard deviation throughout the manuscript. The protocol was conducted as a pilot study with the expectation to be used to power future studies in the lab. Based on prior studies (Goss et al. 2018) in the preterm population with impaired stroke volume augmentation during exercise, a sample size of 8 per group would permit an 80% power to detect a difference in stroke volume increase from rest to maximal exercise, with an alpha level of 0.05.

Group differences in anthropometric characteristics, pulmonary function, and percent change from rest to 30 W in all variables were checked by parametric or non‐parametric unpaired *t*‐test. A two‐way (group × time) mixed‐effects ANOVA was performed to compare the ventilatory, cardiac, and hemodynamic responses at rest and during submaximal and maximal exercise intensity. Sidak post hoc corrections were examined in the event of significant main or interaction effects. All *p*‐values are two‐tailed, and *p* < 0.05 was considered to be statistically significant. Statistical analyses were performed using Prism v.10 (GraphPad Software, San Diego, CA, USA).

## 4 Results

Participants' physical characteristics and resting pulmonary function (assessed by body plethysmography) are shown in Table 1. Gestational age (preterm: 30 ± 3 vs. term: 40 ± 0 weeks, *p* < 0.001) and birth weight (preterm: 1402 ± 320 vs. term: 3331 ± 355 g, *p* < 0.001) were lower in preterm compared to term‐born adults. Self‐reported physical activity level was not different between groups (preterm: 7027 ± 7939 vs. term: 2482 ± 2923 min/week, *p* = 0.174). Cardiorespiratory responses and hemodynamics during the graded exercise protocol are reported in Table 2.

**TABLE 1:** Participants' characteristics and resting pulmonary function and volumes.

	Term	Preterm	*p*
*N*	8	10	
Females	3	6	0.343
Age, years	29 ± 5	30 ± 5	0.721
Height, cm	176 ± 9	161 ± 7	**< 0.001**
Weight, kg	79.2 ± 14.9	70.0 ± 18.1	0.274
BMI, kg/m^2^	25.4 ± 4.6	27.0 ± 6.3	0.772
BSA, m^2^	2.0 ± 0.2	1.8 ± 0.3	0.056
Gestational age, weeks	40 ± 0	30 ± 3	**< 0.001**
Birth weight, g	3331 ± 355	1402 ± 320	**< 0.001**
LVEF, %	61 ± 4	59 ± 5	0.336
MET, min/week	2482 ± 2923	7027 ± 7939	0.174
*Pulmonary function*			
FVC, L	5.05 ± 0.73	3.60 ± 0.83	**0.001**
FVC, % predicted	109 ± 8	100 ± 19	0.227
FEV_1_, L	4.08 ± 0.58	2.84 ± 0.64	**< 0.001**
FEV_1_, % predicted	105 ± 14	95 ± 19	0.221
FEV_1_/FVC, %	81 ± 8	79 ± 7	0.589
FEV_1_/FVC, % predicted	96 ± 7	95 ± 5	0.731
FEF_25%–75%_, L/s	3.98 ± 1.20	3.15 ± 1.01	0.141
FEF_25%–75%_, % predicted	97 ± 31	85 ± 28	0.412
FEF_25%_, L/s	7.47 ± 1.28	5.80 ± 1.94	**0.008**
FEF_25%_, % predicted	98 ± 16	89 ± 26	0.525
FEF_50%_, L/s	4.51 ± 1.18	3.54 ± 1.13	0.102
FEF_50%_, % predicted	88 ± 27	78 ± 25	0.446
FEF_75%_, L/s	1.98 ± 1.04	1.28 ± 0.44	0.093
FEF_75%_, % predicted	105 ± 46	87 ± 34	0.361
PEF, L/s	9.69 ± 1.88	6.54 ± 1.79	**0.002**
PEF, % predicted	108 ± 10	88 ± 20	**0.019**
FET_25%–75%_, s	0.69 ± 0.22	0.63 ± 0.17	0.541
FET_100%_, s	8.40 ± 2.68	9.19 ± 1.62	0.467
PIF, L/s	7.17 ± 1.28	5.17 ± 1.86	**0.020**
VC, L	5.08 ± 0.72	3.62 ± 0.87	**0.002**
VC, % predicted	99 ± 9	90 ± 17	0.237
IC, L	3.59 ± 0.94	2.74 ± 0.93	0.091
IC, % predicted	106 ± 26	103 ± 32	0.851
ERV, L	1.56 ± 0.61	0.93 ± 0.22	**0.012**
ERV, % predicted	96 ± 45	75 ± 18	0.221
V̇_E_, L/min	12.2 ± 4.3	16.7 ± 9.88	0.456
V̇_E_, % predicted	108 ± 28	156 ± 87	0.170
Vt, L	0.994 ± 0.342	0.885 ± 0.445	0.594
Vt, % predicted	173 ± 46	190 ± 120	0.625
Breath frequency, breath/min	13 ± 2	21 ± 8	**0.012**

*Note:* Data are presented as mean ± standard deviation and were compared via independent *t*‐test. Bold values—statistical significance for all < 0.05 or none.

Abbreviations: BMI, body mass index; BSA, body surface area; ERV, expiratory reserve volume; FEF, forced expiratory flow (25%, 50%, and 75% indicate the flow corresponding to 25%, 50%, and 75% of total lung volume); FET, forced expiratory time; FEV_1_, forced expiratory volume in 1 s; FVC, forced vital capacity; IC, inspiratory capacity; LVEF, left ventricle ejection fraction; PEF, peak expiratory flow; PIF, peak inspiratory flow; VC, vital capacity; V̇_E_, pulmonary ventilation; Vt, tidal volume.

**TABLE 2:** Cardiopulmonary responses and hemodynamics at rest and during incremental cycling exercise in term and preterm born adults.

Variable	Rest	30 W	60 W	Peak	Group	Stage	Group × Stage
Power output, Watts							
Term	—	—	—	208 ± 69	*p* < 0.001	—	—
Preterm	—	—	—	108 ± 18^a^			
V̇O_2_, L/min							
Term	0.29 ± 0.07	0.83 ± 0.09	1.14 ± 0.09	2.52 ± 0.85	*p* = 0.023	*p* < 0.001	*p* < 0.001
Preterm	0.23 ± 0.05	0.85 ± 0.16	1.14 ± 0.16	1.58 ± 0.29^a^			
V̇O_2_, mL/kg/min							
Term	3.7 ± 0.7	10.7 ± 1.7	14.8 ± 2.5	31.9 ± 9.1	*p* = 0.791	*p* < 0.001	*p* < 0.001
Preterm	3.5 ± 1.0	13.3 ± 3.7	17.8 ± 4.9	24.7 ± 7.0			
V̇O_2_, % predicted							
Term	—	—	—	77 ± 21	*p* = 0.132	—	—
Preterm	—	—	—	62 ± 18			
V̇CO_2_, L/min							
Term	0.23 ± 0.07	0.70 ± 0.08	1.04 ± 0.07	3.00 ± 0.94	*p* = 0.015	*p* < 0.001	*p* < 0.001
Preterm	0.20 ± 0.06	0.79 ± 0.14	1.12 ± 0.13	1.82 ± 0.40^a^			
V̇_E_, L/min							
Term	10.5 ± 1.9	23.0 ± 3.3	32.0 ± 3.1	113.7 ± 35.4	*p* = 0.051	*p* < 0.001	*p* < 0.001
Preterm	12.2 ± 4.5	28.9 ± 5.9^a^	38.7 ± 3.9^a^	68.5 ± 18.5^a^			
Respiratory rate, breath/min							
Term	14 ± 3	16 ± 4	19 ± 3	46 ± 10	*p* = 0.180	***p*** < **0.001**	***p*** = **0.001**
Preterm	18 ± 4	23 ± 9^a^	29 ± 8^a^	42 ± 11			
Tidal volume, L							
Term	0.763 ± 0.159	1.505 ± 0.265	1.702 ± 0.280	2.448 ± 0.509	*p* = 0.036	*p* < 0.001	*p* < 0.001
Preterm	0.713 ± 0.245	1.361 ± 0.413	1.402 ± 0.314^a^	1.666 ± 0.451^a^			
Respiratory exchange ratio							
Term	0.76 ± 0.05	0.83 ± 0.07	0.92 ± 0.07	1.20 ± 0.07	*p* = 0.042	*p* < 0.001	*p* = 0.020
Preterm	0.86 ± 0.17	0.93 ± 0.03^a^	0.99 ± 0.07	1.15 ± 0.07			
P_ET_CO_2_, mmHg							
Term	40 ± 4	43 ± 5	44 ± 3	34 ± 4	*p* = 0.532	***p*** < **0.001**	*p* = 0.097
Preterm	38 ± 9	40 ± 6	41 ± 6	36 ± 6			
Pulse oxygen saturation, %							
Term	98 ± 3	98 ± 4	97 ± 4	98 ± 2	*p* = 0.148	*p* = 0.680	*p* = 0.559
Preterm	99 ± 1	99 ± 1	99 ± 1	99 ± 1			
RPE							
Term	—	8 ± 1	10 ± 1	18 ± 1	*p* = 0.111	*p* < 0.001	*p* < 0.001
Preterm	—	10 ± 3	14 ± 3^a^	18 ± 2			
RPB							
Term	0 ± 0	1 ± 0	1 ± 1	7 ± 2	*p* = 0.057	*p* < 0.001	*p* = 0.012
Preterm	0 ± 0	2 ± 2^a^	4 ± 3^a^	7 ± 3			
Heart rate, beats/min							
Term	80 ± 17	99 ± 19	118 ± 20	185 ± 8	*p* = 0.608	***p*** < **0.001**	***p*** = **0.002**
Preterm	79 ± 10	109 ± 14	134 ± 20	175 ± 16			
Heart rate (% predicted)							
Term	—	—	—	98 ± 4	*p* = 0.097	—	—
Preterm	—	—	—	93 ± 9			
Systolic BP, mmHg							
Term	119 ± 12	138 ± 17	151 ± 19	185 ± 33	*p* = 0.417	*p* < 0.001	*p* = 0.605
Preterm	119 ± 17	144 ± 22	167 ± 14	188 ± 17			
Diastolic BP, mmHg							
Term	83 ± 9	78 ± 14	76 ± 11	81 ± 14	*p* = 0.977	*p* = 0.382	*p* = 0.035
Preterm	70 ± 13^a^	80 ± 15	87 ± 14	83 ± 21			
Cardiac index, L/min/m^2^							
Term	2.5 ± 0.6	4.6 ± 0.7	5.6 ± 0.6	8.9 ± 1.6	*p* = 0.699	***p*** < **0.001**	***p*** < **0.001**
Preterm	2.7 ± 0.6	5.1 ± 0.7	6.0 ± 0.8	7.5 ± 1.0			
Stroke volume index, mL/m^2^							
Term	33 ± 10	48 ± 12	49 ± 10	48 ± 11	*p* = 0.542	***p*** < **0.001**	*p* = 0.061
Preterm	35 ± 7	47 ± 7	45 ± 7	42 ± 7			
TPR, mmHg/L/min							
Term	19.6 ± 3.2	11.0 ± 1.9	9.2 ± 1.7	6.9 ± 2.1	*p* = 0.257	***p*** < **0.001**	*p* = 0.248
Preterm	19.1 ± 4.6	11.8 ± 1.3	11.1 ± 1.4^a^	9.6 ± 1.6^a^			
Blood lactate, mmol/L							
Term	1.3 ± 0.4	1.7 ± 0.8	2.5 ± 1.4	11.4 ± 2.3	*p* = 0.564	***p*** < **0.001**	***p*** < **0.001**
Preterm	1.5 ± 0.9	2.4 ± 1.0	4.0 ± 1.1^a^	8.1 ± 2.0^a^			
a‐*v*O_2_, mL/dL							
Term	5.9 ± 1.1	9.4 ± 1.5	10.5 ± 0.8	14.1 ± 1.6	*p* = 0.630	***p*** < **0.001**	***p*** = **0.002**
Preterm	4.9 ± 1.1	9.9 ± 1.3	11.3 ± 2.0	12.6 ± 1.7			

*Note:* Data are presented as mean ± standard deviation and were compared via two‐way (group × stage) ANOVA. Peak power output and % predicted values for V̇O_2_ and heart rate were compared via independent *t*‐test. Bold values—statistical significance for all < 0.05 or none.

Abbreviations: a‐*v*O_2_diff, arterial–venous oxygen difference; BP, blood pressure; P_ET_CO_2_, end‐tidal carbon dioxide partial pressure; RER, respiratory exchange ratio; RPB, rate of perceived breathlessness; RPE, rate of perceived exertion; TPR, total peripheral resistance; V̇CO_2_, carbon dioxide production; V̇_E_, pulmonary ventilation; V̇O_2_, oxygen uptake.

^a^ Represents between‐group difference.

**FIGURE 1:**
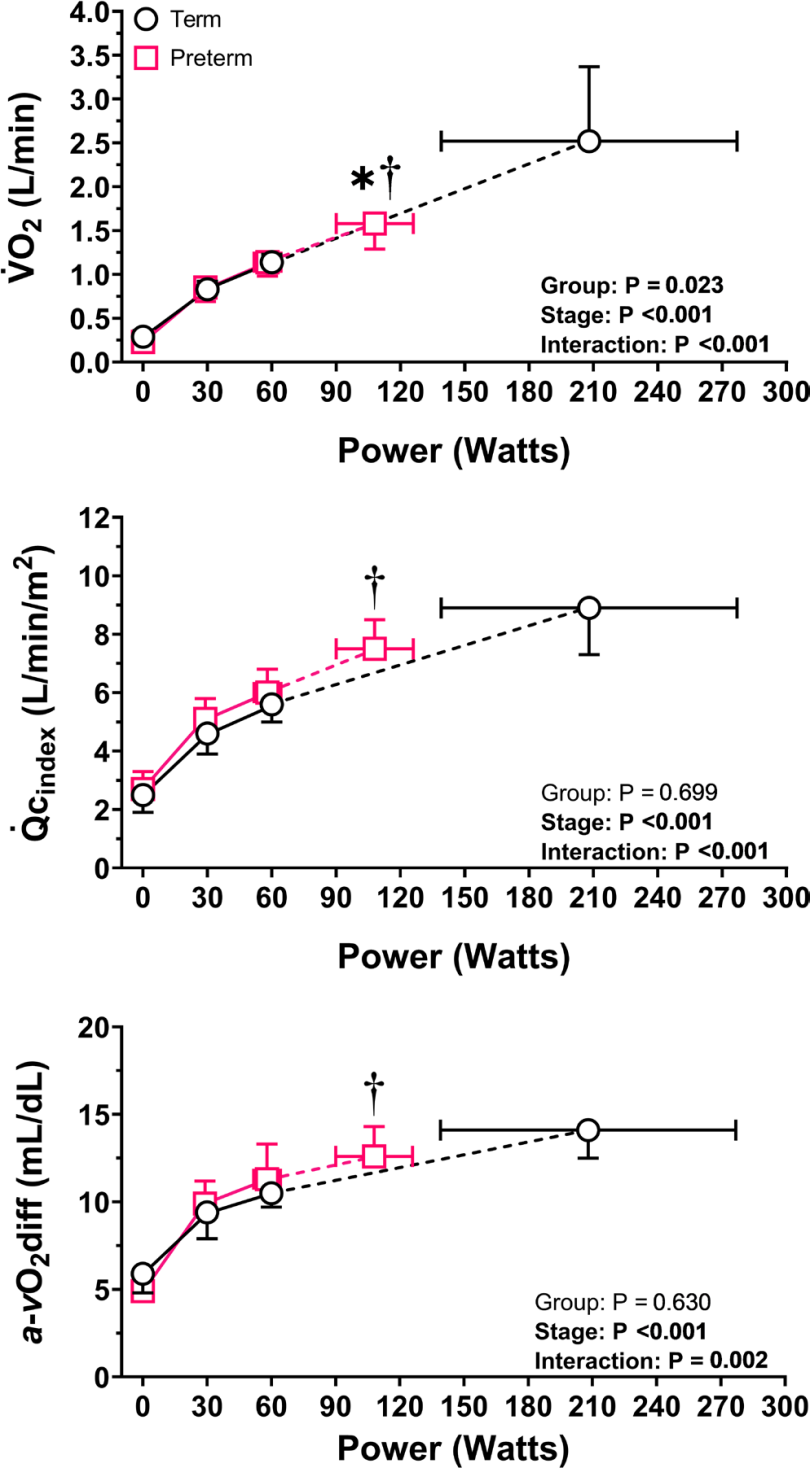
Oxygen uptake (V̇O_2_, top panel), cardiac index (Q̇c_index_, middle panel), and arterial–venous oxygen difference (a‐*v*O_2_diff, bottom panel) kinetics at rest, 30 W, 60 W, and peak exercise intensity in preterm (pink squares) and term (black circles) born adults. *p < 0.05 from term versus preterm born adults for *y*‐axis comparison; †*p* < 0.05 from term versus preterm born adults for *x*‐axis comparison.

**FIGURE 2:**
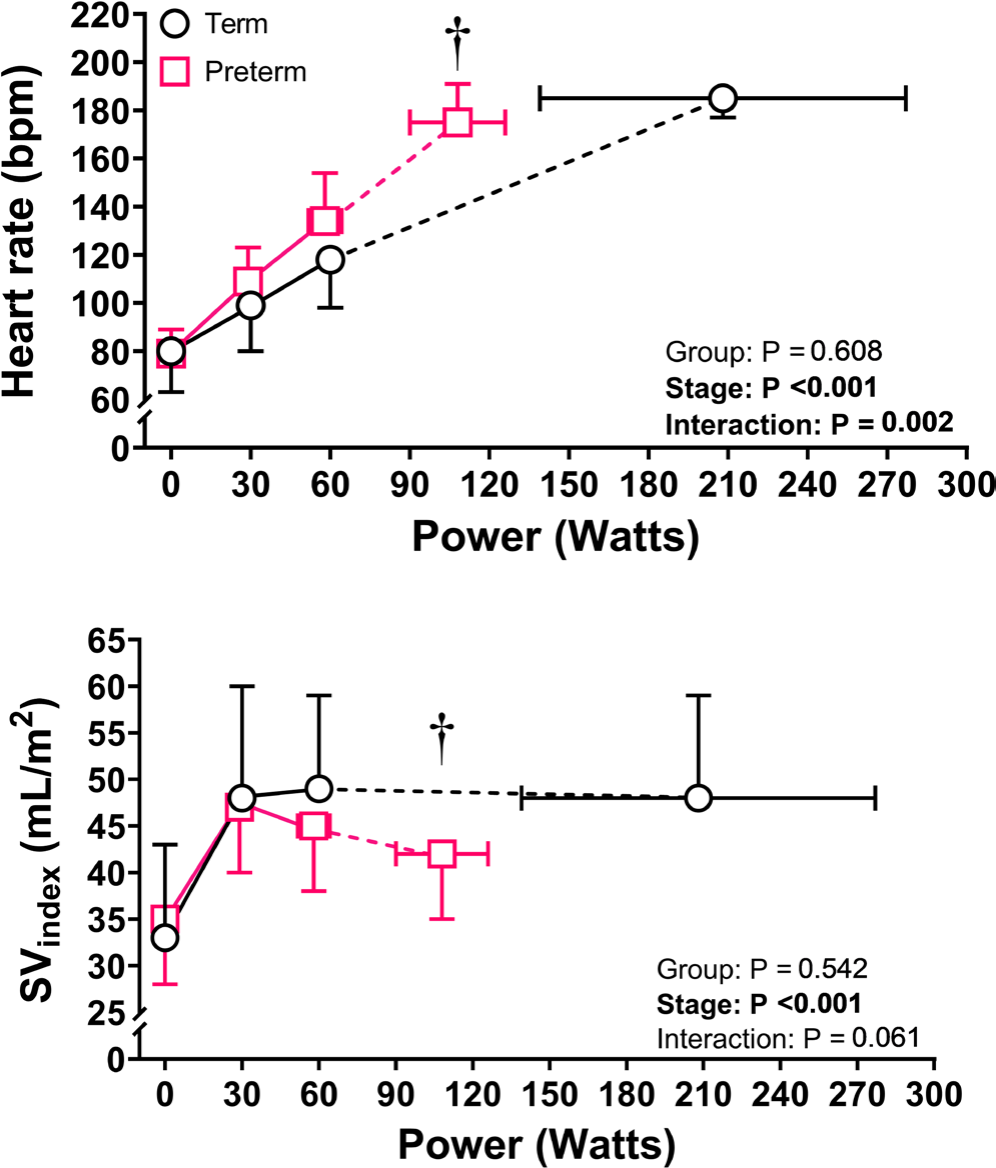
Heart rate (top panel), and stroke volume index (SV_index_, bottom panel) kinetics at rest, 30 W, 60 W, and peak exercise intensity in preterm (pink squares) and term (black circles) born adults. †*p* < 0.05 from term versus preterm born adults for *x*‐axis comparison.

**FIGURE 3:**
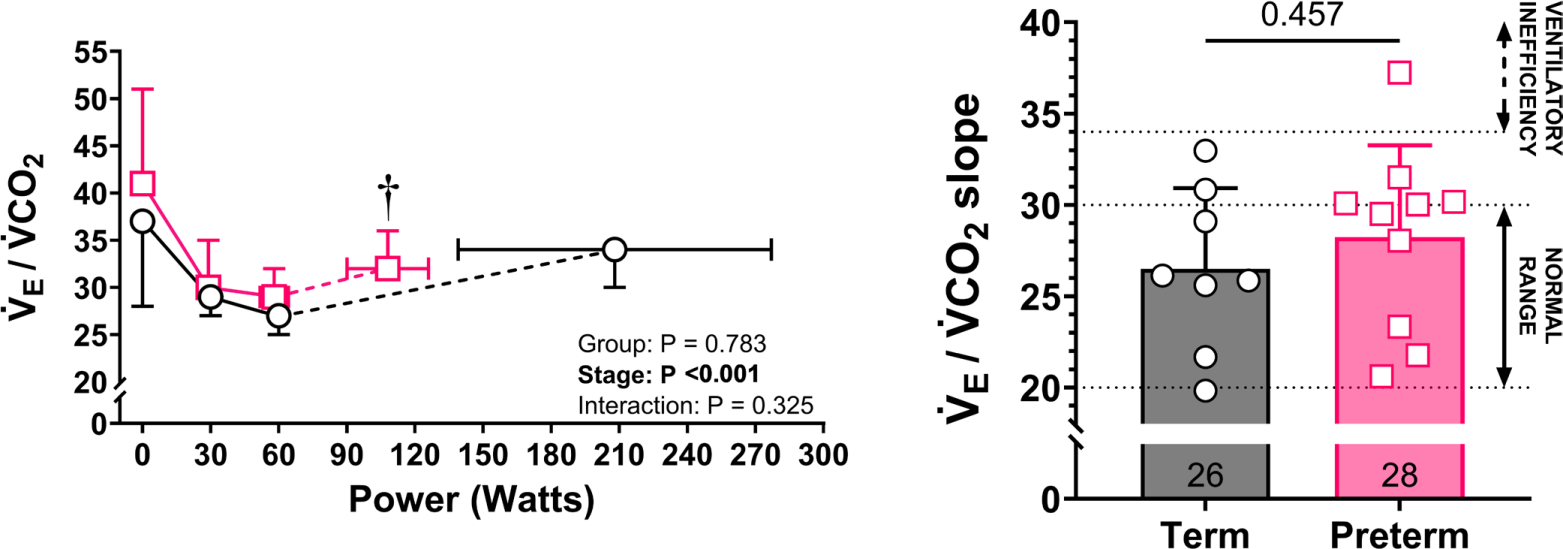
Pulmonary ventilation versus carbon dioxide production (V̇_E_/V̇CO_2_) slope (left panel) kinetics at rest, 30 W, 60 W, and peak exercise intensity in preterm (pink squares) and term (black circles) born adults. Right panel shows group means (±standard deviation) and individual values for the V̇_E_/V̇CO_2_ slope in preterm (pink squares) and term (black circles) born adults. The mean V̇_E_/V̇CO_2_ slope for each group is reported inside each bar at the bottom. †*p* < 0.05 from term versus preterm born adults for *x*‐axis comparison.

At rest, pulmonary function, lung volumes, heart rate, Q̇c_index_, and a‐*v*O_2_diff were not different between preterm and term‐born adults. Figure 1 shows the oxygen uptake, cardiac index, and oxygen extraction responses to submaximal (30 and 60 W) and maximal exercise intensity, while the heart rate and stroke volume index kinetics are shown in Figure 2. V̇O_2_, heart rate, Q̇c_index_, and a‐*v*O_2_diff increased similarly from rest to 30 and 60 W in both groups. The V̇_E_/V̇CO_2_ slope (preterm: 28 ± 5 vs. term: 26 ± 4, *p* = 0.457; Figure 3) and stroke volume reserve (preterm: 38% ± 19% vs. term: 48% ± 20%, *p* = 0.296) were also not different between groups. At both 30 W and 60 W, preterm adults had higher V̇_E_ compared to term‐born peers (30 W: 28.9 ± 5.9 vs. 23.0 ± 3.3, *p* = 0.019; 60 W: 38.7 ± 3.9 vs. 32.0 ± 3.1, *p* < 0.001), which is reflective of a higher relative exercise intensity in the preterm cohort. TPR (Table 2) decreased similarly in both groups from rest to 30 W exercise, while at 60 W preterm adults showed higher TPR compared to term‐born peers (11.1 ± 1.4 vs. 9.2 ± 1.7 mmHg/L/min, *p* = 0.038).

**FIGURE 4:**
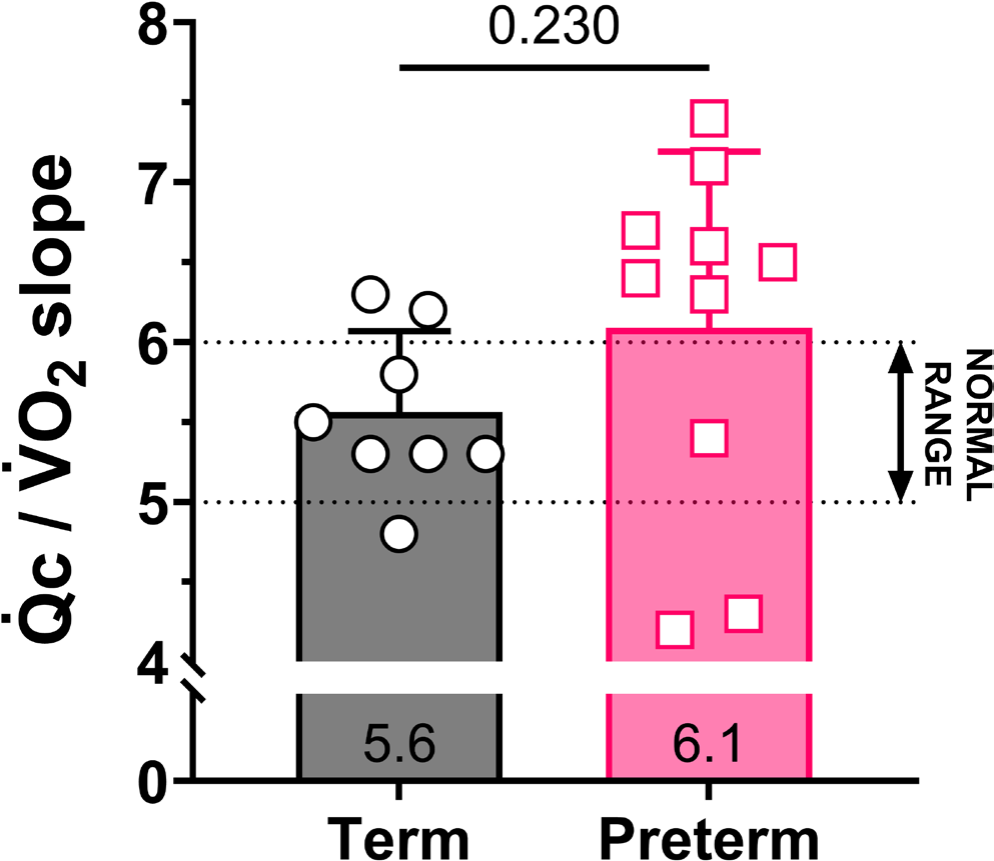
Group means (±standard deviation) and individual values for the cardiac output‐oxygen uptake (Q̇c/V̇O_2_) slope in preterm (pink squares) and term (black circles) born adults. The mean Q̇c/V̇O_2_ slope for each group is reported inside each bar at the bottom.

At peak exercise intensity, compared to term born peers, preterm adults showed lower power output (108 ± 18 vs. 208 ± 69 W, *p* < 0.001), absolute V̇O_2_ (1.58 ± 0.29 vs. 2.52 ± 0.85 L/min, *p* = 0.017), V̇_E_ (68.5 ± 18.5 vs. 113.7 ± 35.4 L/min, *p* = 0.008), and Q̇c_index_ (7.5 ± 1.0 vs. 8.9 ± 1.6 L/min/m^2^, *p* = 0.057). Relative peak V̇O_2_ (31.9 ± 9.1 vs. 24.7 ± 7.0 mL/kg/min, *p* = 0.089) and peak HR were similar between groups (preterm: 175 ± 16 vs. term: 185 ± 8 bpm, *p* = 0.104). The increase in stroke volume index from rest to peak exercise intensity was blunted in preterm compared to term (8 ± 7 vs. 15 ± 6 mL/m^2^, *p* = 0.032) born adults. Peak a‐*v*O_2_diff (preterm: 12.6 ± 1.7 vs. term: 14.1 ± 1.6 mL/dL, *p* = 0.096) and the Q̇c‐V̇O_2_ slope (preterm: 6.1 ± 1.1 vs. term: 5.6 ± 0.5, *p* = 0.230; Figure 4) were not different between groups, though there was greater variability in Q̇c‐V̇O_2_ slope in preterm born adults. TPR was higher at peak exercise in preterm compared to term born participants (9.6 ± 1.6 vs. 6.9 ± 2.1 mmHg/L/min, *p* = 0.013).

Blood lactate concentration was not different between groups at rest and 30 W exercise (Table 2). The preterm cohort showed greater blood lactate concentration at 60 W compared to term‐born peers (4.0 ± 1.1 vs. 2.5 ± 1.4 mmol/L, *p* = 0.044), while at peak exercise blood lactate was significantly higher in term compared to preterm‐ born adults (11.4 ± 2.3 vs. 8.1 ± 2.0 mmol/L, *p* = 0.009) adults. The oxygen cost of cycling was not different between groups (preterm: 9.1 ± 1.6 vs. term: 9.3 ± 1.1 mL/min/W, *p* = 0.779).

## 5 Discussion

This study aimed to investigate the central mechanisms of reduced exercise capacity in prematurely born but otherwise healthy adults who reported exertional dyspnea despite normal resting cardiopulmonary function, and was the first study to evaluate the cardiac response to incremental exercise using a highly accurate, acetylene rebreathe technique in preterm born adults. The main findings are (1) adults born preterm present with lower Q̇c at maximal, but not submaximal, exercise intensity, (2) impaired stroke volume augmentation in response to incremental exercise intensity underlies the lower cardiac output response to exercise in preterm adults compared to age‐matched individuals born at term, and (3) the greater variability of the Q̇c‐V̇O_2_ slope in prematurely born adults may suggest two distinct phenotypes of exercise intolerance (centrally limited: Q̇c‐V̇O_2_ < 5 vs. peripherally limited: Q̇c‐V̇O_2_ > 6 (Proctor et al. 1998)) in this cohort.

An appropriate systemic response to exercise requires the integration of multiple physiological systems including the respiratory, cardiovascular, and musculoskeletal/neuromuscular systems. Any impairment at these levels can negatively impact exercise capacity, including mechanical ventilatory constraints, impaired pulmonary gas exchange, decreased pulmonary vascular capacity, reduced cardiac function, impaired muscle oxygen uptake/utilization and/or neuromuscular function. Premature birth severely hinders the normal development of organs and systems (Luu et al. 2017), leading to incomplete maturation during adulthood (Telles et al. 2020; Bates et al. 2020) and low exercise capacity. While the key contributor(s) to reduced exercise capacity in healthy adults born preterm remain debated, impaired cardiac function rather than altered pulmonary function seems to explain the lower exercise capacity in this population, at least for those with normal resting lung function (Huckstep et al. 2021). Even less data is available on the role of skeletal muscle oxygen extraction in adults born preterm (Manferdelli, Narang, Bourdillon, et al. 2023; Owen‐Jones et al. 2020).

The initial step in the oxygen transport from ambient air to skeletal muscle mitochondria is represented by pulmonary ventilation and gas exchange efficiency (Wagner 1988, 1991). Previous studies investigating the ventilatory limitation to exercise in preterm born children and adults suggest lower peak V̇_E_ (Duke et al. 2018, 2019; Narang et al. 2022), despite similar alveolar‐arterial oxygen difference (Lovering et al. 1985; Duke et al. 2014; Farrell et al. 2015). Mechanical ventilatory constraints may also play a role in exercise intolerance in this population, as heliox breathing was shown to improve exercise capacity in preterm born adults (Duke et al. 2019). However, in this latter study, the preterm cohort had lower resting pulmonary function compared to the control group born at term, and about ~50% had bronchopulmonary dysplasia. In the present study, we investigated prematurely born adults with normal resting lung function, and therefore it is not surprising that the ventilatory response to exercise was similar to term born adults. Moreover, our preterm cohort showed normal gas exchange in response to exercise, as demonstrated by similar V̇_E_/V̇CO_2_ slope between groups. A steeper V̇_E_/V̇CO_2_ slope (> 34) is typical of a wide range of conditions characterized by ventilation‐to‐perfusion mismatch, including heart failure (Lewis et al. 2008), pulmonary vascular disease, and pulmonary hypertension (Sun et al. 2001). However, the present preterm born cohort was screened for pulmonary hypertension using echocardiography, and therefore it is not surprising that the V̇_E_/V̇CO_2_ slope was similar to their term born peers. Interestingly, the majority of our preterm born adults present with a V̇_E_/V̇CO_2_ slope right on the upper limit of the normal range. Although this may indicate subclinical pulmonary vascular disease, it is unlikely that pulmonary hypertension contributed to exercise limitation in the present preterm cohort.

Limited literature has focused on prematurity‐induced changes in cardiac structure and function and their impact on exercise capacity (Telles et al. 2020; Schuermans and Lewandowski 2022). Smaller biventricular size and mass (Barton et al. 2024; Blair et al. 2025), and reduced LV longitudinal systolic, diastolic, and rotational function at rest and during exercise have been reported in preterm adolescents and adults (Barton et al. 2024; Lewandowski, Bradlow, et al. 2013; Lewandowski, Augustine, et al. 2013). Importantly, these cardiac impairments are exaggerated in the right ventricle (Macdonald et al. 2021; Lewandowski, Bradlow, et al. 2013). Using stress echocardiography, Huckstep and colleagues showed a drop in LVEF during exercise in preterm adults, while no change was observed in the control group born at term (Huckstep et al. 2018). Similarly, previous studies using thoracic bioimpedance reported a progressive decline in stroke volume with increasing exercise intensity in preterm born individuals, despite a normal response at the onset of exercise (Haraldsdottir et al. 2020; Manferdelli, Narang, Bourdillon, et al. 2023; Huckstep et al. 2021). Our results confirm these previous findings of impaired cardiac output response in healthy prematurely born adults, which is mediated by a significant decrease in stroke volume when exercise intensity increases above moderate intensity. Notably, the present study was the first to accurately measure (using acetylene rebreathe method) cardiac output and stroke volume during incremental exercise in adults born prematurely. Whether the decline in stroke volume with increasing exercise intensity above 30 W is mediated by an exaggerated decrease in end‐diastolic volume, rather than an increase in end‐systolic volume, remains to be investigated in future studies combining cardiac imaging techniques and exercise in this cohort.

The final step in the oxygen cascade is represented by oxygen diffusion, extraction, and utilization at the skeletal muscle level (Wagner 1991). However, previous studies investigating the mechanisms of exercise limitation in prematurely born adults have primarily focused on central mechanisms (i.e., pulmonary and cardiac) of oxygen transport during exercise, with less attention to the contribution of peripheral factors (skeletal muscle). Existing findings are primarily based on animal models using postnatal oxygen supplementation therapy to simulate premature birth. Premature birth elicits a greater fat‐to‐lean mass ratio, muscle fatigability, inflammation and atrophy, fiber type shifting (from type I slow fibers to type IIb fast‐fatigable fibers), and lower skeletal muscle mitochondrial oxidative capacity (Tetri et al. 2018; Deprez et al. 2021). Notably, these negative effects were more pronounced in preterm male rodents compared to females (Tetri et al. 2018). In preterm humans, normal skeletal muscle microvascular reactive hyperemia (Manferdelli, Narang, Pialoux, et al. 2023), lower skeletal muscle contractility in both upper (Barton et al. 2024) and lower limbs (Rogers et al. 2005), and increased oxygen extraction (assessed non‐invasively as greater NIRS‐derived deoxygenated hemoglobin) during submaximal (Owen‐Jones et al. 2020) and maximal (Manferdelli, Narang, Bourdillon, et al. 2023) exercise were observed in preterm children, adolescents, and adults compared to age‐matched peers born at term. In the present study, preterm adults exhibited higher total peripheral resistance at submaximal (60 W) and maximal exercise intensity, suggesting that impairments in exercise‐induced skeletal muscle vasodilation and/or functional sympatholysis may also contribute to limiting exercise capacity in this cohort.

Finally, the analyses of the Q̇c‐V̇O_2_ slope in the present study may suggest two distinct phenotypes of exercise limitation in preterm born adults. In healthy adults, Q̇c increases 5–6 L/min per unit increase in V̇O_2_ (Julius et al. 1967), with this relationship being maintained across age, gender, and training status (Proctor et al. 1998). On the contrary, a Q̇c‐V̇O_2_ slope lower than 5 or higher than 6 indicates a primarily central or peripheral limitation to exercise capacity, respectively. While the majority of existing literature points to primarily central (either pulmonary or cardiac) mechanisms of exercise intolerance in preterm adults (Goss et al. 2020; Haraldsdottir et al. 2018, 2020; Manferdelli, Narang, Bourdillon, et al. 2023; Huckstep et al. 2018, 2021; Barton et al. 2024; Duke et al. 2019), 70% of the preterm cohort studied presented with a Q̇c‐V̇O_2_ slope > 6, which suggests that peripheral, rather than central, mechanisms contribute to exercise limitations in these individuals. Our results suggest that additional work investigating the skeletal muscle/vascular/mitochondrial (peripheral) unit is required to confirm our findings.

Our findings add to the existing literature by identifying a significant contribution of both impaired stroke volume augmentation from rest to peak exercise intensity and reduced oxygen extraction/utilization at the skeletal muscle level in limiting exercise capacity in preterm adults compared to term‐born peers.

## 6 Conclusions

Prematurely born adults with normal resting cardiopulmonary function and exertional dyspnea present with lower exercise capacity, as demonstrated by lower peak power output at exhaustion and V̇O_2peak_. Impaired stroke volume augmentation primarily underlies the reduced exercise capacity in preterm born adults with normal resting pulmonary function compared to age‐matched individuals born at term. However, peripheral mechanisms of exercise limitation may also contribute to reduced exercise capacity in preterm individuals. Future studies should investigate the cardiac mechanisms of impaired stroke volume augmentation in this population. Moreover, more research is needed to expand upon our findings of the contribution of skeletal muscle and mitochondrial function/morphology to impaired exercise capacity in adults born preterm.

## Conflicts of Interest

The authors declare no conflicts of interest.

## Data Availability

The data that support the findings of this study are available from the corresponding author upon reasonable request.

## References

[cph470049-bib-0001] Ahmed, A. M. , S. M. Grandi , E. Pullenayegum , et al. 2024. “Short‐Term and Long‐Term Mortality Risk After Preterm Birth.” JAMA Network Open 7: e2445871. 39565625 10.1001/jamanetworkopen.2024.45871 PMC11579792

[cph470049-bib-0002] Barton, G. P. , A. Chandra , N. Sanchez‐Solano , J. D. Berry , and K. N. Goss . 2024. “Smaller Left Ventricular Size but Preserved Function in Adolescents and Adults Born Preterm.” Journal of the American Heart Association 13: e035529. 39248261 10.1161/JAHA.124.035529 PMC11935619

[cph470049-bib-0003] Bates, M. L. , P. T. Levy , A. M. Nuyt , K. N. Goss , A. J. Lewandowski , and P. J. McNamara . 2020. “Adult Cardiovascular Health Risk and Cardiovascular Phenotypes of Prematurity.” Journal of Pediatrics 227: 17–30. 32931771 10.1016/j.jpeds.2020.09.019

[cph470049-bib-0004] Blair, Z. W. , A. Chandra , G. P. Barton , N. S. Solano , J. D. Berry , and K. N. Goss . 2025. “Reduced Right Ventricular Size and Function in Adolescents and Adults Born Preterm.” Journal of the American Society of Echocardiography 38: 615–623. 40252713 10.1016/j.echo.2025.04.007

[cph470049-bib-0005] Borg, G. A. 1982. “Psychophysical Bases of Perceived Exertion.” Medicine and Science in Sports and Exercise 14: 377–381. 7154893

[cph470049-bib-0006] Deprez, A. , Z. Orfi , A. Radu , et al. 2021. “Transient Neonatal Exposure to Hyperoxia, an Experimental Model of Preterm Birth, Leads to Skeletal Muscle Atrophy and Fiber Type Switching.” Clinical Science 135: 2589–2605. 34750633 10.1042/CS20210894

[cph470049-bib-0007] Duke, J. W. , J. E. Elliott , S. S. Laurie , et al. 2014. “Pulmonary Gas Exchange Efficiency During Exercise Breathing Normoxic and Hypoxic Gas in Adults Born Very Preterm With Low Diffusion Capacity.” Journal of Applied Physiology (1985) 117, no. 5: 473–481. 10.1152/japplphysiol.00307.2014 24970854

[cph470049-bib-0008] Duke, J. W. , I. M. Gladstone , A. W. Sheel , and A. T. Lovering . 2018. “Premature Birth Affects the Degree of Airway Dysanapsis and Mechanical Ventilatory Constraints.” Experimental Physiology 103: 261–275. 29193495 10.1113/EP086588

[cph470049-bib-0009] Duke, J. W. , A. J. Lewandowski , S. H. Abman , and A. T. Lovering . 2022. “Physiological Aspects of Cardiopulmonary Dysanapsis on Exercise in Adults Born Preterm.” Journal of Physiology 600: 463–482. 34961925 10.1113/JP281848 PMC9036864

[cph470049-bib-0010] Duke, J. W. , A. M. Zidron , I. M. Gladstone , and A. T. Lovering . 2019. “Alleviating Mechanical Constraints to Ventilation With Heliox Improves Exercise Endurance in Adult Survivors of Very Preterm Birth.” Thorax 74: 302–304. 30217953 10.1136/thoraxjnl-2018-212346

[cph470049-bib-0011] Farrell, E. T. , M. L. Bates , D. F. Pegelow , et al. 2015. “Pulmonary Gas Exchange and Exercise Capacity in Adults Born Preterm.” Annals of the American Thoracic Society 12: 1130–1137. 26053283 10.1513/AnnalsATS.201410-470OC PMC4566409

[cph470049-bib-0012] Goss, K. N. , E. D. Austin , T. J. Battiola , R. S. Tepper , and T. Lahm . 2019. “Novel Early Life Risk Factors for Adult Pulmonary Hypertension.” Pulmonary Circulation 9: 2045894019845615. 10.1177/2045894019842002 PMC6469274 30880574

[cph470049-bib-0013] Goss, K. N. , A. G. Beshish , G. P. Barton , et al. 2018. “Early Pulmonary Vascular Disease in Young Adults Born Preterm.” American Journal of Respiratory and Critical Care Medicine 198: 1549–1558. 29944842 10.1164/rccm.201710-2016OC PMC6298636

[cph470049-bib-0014] Goss, K. N. , K. Haraldsdottir , A. G. Beshish , et al. 2020. “Association Between Preterm Birth and Arrested Cardiac Growth in Adolescents and Young Adults.” JAMA Cardiology 5: 910–919. 32432648 10.1001/jamacardio.2020.1511 PMC7240643

[cph470049-bib-0015] Graham, B. L. , I. Steenbruggen , M. R. Miller , et al. 2019. “Standardization of Spirometry 2019 Update. An Official American Thoracic Society and European Respiratory Society Technical Statement.” American Journal of Respiratory and Critical Care Medicine 200: e70–e88. 31613151 10.1164/rccm.201908-1590ST PMC6794117

[cph470049-bib-0016] Hankinson, J. L. , J. R. Odencrantz , and K. B. Fedan . 1999. “Spirometric Reference Values From a Sample of the General U.S. Population.” American Journal of Respiratory and Critical Care Medicine 159: 179–187. 9872837 10.1164/ajrccm.159.1.9712108

[cph470049-bib-0017] Haraldsdottir, K. , A. M. Watson , D. F. Pegelow , et al. 2020. “Blunted Cardiac Output Response to Exercise in Adolescents Born Preterm.” European Journal of Applied Physiology 120: 2547–2554. 32862247 10.1007/s00421-020-04480-9 PMC12908129

[cph470049-bib-0018] Haraldsdottir, K. , O. Wieben , G. Barton , K. Goss , A. Watson , and M. Eldridge . 2018. “Exercise Intolerance in Adolescents Born Preterm Associated With Left Ventricular Diastolic Dysfunction: Experimental Biology 2018 Meeting.” FASEB Journal 32: 853‐817.

[cph470049-bib-0019] Hardin, E. A. , D. Stoller , J. Lawley , et al. 2020. “Noninvasive Assessment of Cardiac Output: Accuracy and Precision of the Closed‐Circuit Acetylene Rebreathing Technique for Cardiac Output Measurement.” Journal of the American Heart Association 9: e015794. 32851906 10.1161/JAHA.120.015794 PMC7660774

[cph470049-bib-0020] Harris, S. , L. Perston , K. More , et al. 2021. “Cardiac Structure and Function in Very Preterm‐Born Adolescents Compared to Term‐Born Controls: A Longitudinal Cohort Study.” Early Human Development 163: 105505. 34763163 10.1016/j.earlhumdev.2021.105505

[cph470049-bib-0021] Huckstep, O. J. , H. Burchert , W. Williamson , et al. 2021. “Impaired Myocardial Reserve Underlies Reduced Exercise Capacity and Heart Rate Recovery in Preterm‐Born Young Adults.” European Heart Journal Cardiovascular Imaging 22: 572–580. 32301979 10.1093/ehjci/jeaa060 PMC8081423

[cph470049-bib-0022] Huckstep, O. J. , W. Williamson , F. Telles , et al. 2018. “Physiological Stress Elicits Impaired Left Ventricular Function in Preterm‐Born Adults.” Journal of the American College of Cardiology 71: 1347–1356. 29566820 10.1016/j.jacc.2018.01.046 PMC5864965

[cph470049-bib-0023] Jarvis, S. S. , B. D. Levine , G. K. Prisk , et al. 2007. “Simultaneous Determination of the Accuracy and Precision of Closed‐Circuit Cardiac Output Rebreathing Techniques.” Journal of Applied Physiology (1985) 103: 867–874. 10.1152/japplphysiol.01106.2006 17556490

[cph470049-bib-0024] Julius, S. , A. Amery , L. S. Whitlock , and J. Conway . 1967. “Influence of Age on the Hemodynamic Response to Exercise.” Circulation 36: 222–230. 4952658 10.1161/01.cir.36.2.222

[cph470049-bib-0025] Lewandowski, A. J. , D. Augustine , P. Lamata , et al. 2013. “Preterm Heart in Adult Life: Cardiovascular Magnetic Resonance Reveals Distinct Differences in Left Ventricular Mass, Geometry, and Function.” Circulation 127: 197–206. 23224059 10.1161/CIRCULATIONAHA.112.126920

[cph470049-bib-0026] Lewandowski, A. J. , W. M. Bradlow , D. Augustine , et al. 2013. “Right Ventricular Systolic Dysfunction in Young Adults Born Preterm.” Circulation 128: 713–720. 23940387 10.1161/CIRCULATIONAHA.113.002583

[cph470049-bib-0027] Lewis, G. D. , R. V. Shah , P. P. Pappagianopolas , D. M. Systrom , and M. J. Semigran . 2008. “Determinants of Ventilatory Efficiency in Heart Failure: The Role of Right Ventricular Performance and Pulmonary Vascular Tone.” Circulation. Heart Failure 1: 227–233. 19808296 10.1161/CIRCHEARTFAILURE.108.785501 PMC2806812

[cph470049-bib-0028] Liang, X. , Y. Lyu , J. Li , Y. Li , and C. Chi . 2024. “Global, Regional, and National Burden of Preterm Birth, 1990‐2021: A Systematic Analysis From the Global Burden of Disease Study 2021.” EClinicalMedicine 76: 102840. 39386159 10.1016/j.eclinm.2024.102840 PMC11462015

[cph470049-bib-0029] Lovering, A. T. , S. S. Laurie , J. E. Elliott , et al. 1985. “Normal Pulmonary Gas Exchange Efficiency and Absence of Exercise‐Induced Arterial Hypoxemia in Adults With Bronchopulmonary Dysplasia.” Journal of Applied Physiology (1985) 115: 1050–1056. 10.1152/japplphysiol.00592.2013 23869070

[cph470049-bib-0030] Luu, T. M. , M. O. Rehman Mian , and A. M. Nuyt . 2017. “Long‐Term Impact of Preterm Birth: Neurodevelopmental and Physical Health Outcomes.” Clinics in Perinatology 44: 305–314. 28477662 10.1016/j.clp.2017.01.003

[cph470049-bib-0031] Macdonald, J. A. , G. S. Roberts , P. A. Corrado , et al. 2021. “Exercise‐Induced Irregular Right Heart Flow Dynamics in Adolescents and Young Adults Born Preterm.” Journal of Cardiovascular Magnetic Resonance 23: 116. 34670573 10.1186/s12968-021-00816-2 PMC8529801

[cph470049-bib-0032] Manferdelli, G. , B. J. Narang , N. Bourdillon , T. Debevec , and G. P. Millet . 2023. “Physiological Responses to Exercise in Hypoxia in Preterm Adults: Convective and Diffusive Limitations in the O_2_ Transport.” Medicine and Science in Sports and Exercise 55: 482–496. 36459101 10.1249/MSS.0000000000003077

[cph470049-bib-0033] Manferdelli, G. , B. J. Narang , V. Pialoux , G. Giardini , T. Debevec , and G. P. Millet . 2023. “Microvascular and Oxidative Stress Responses to Acute High‐Altitude Exposure in Prematurely Born Adults.” Scientific Reports 13: 6860. 37100885 10.1038/s41598-023-34038-6 PMC10133287

[cph470049-bib-0034] Narang, B. J. , G. Manferdelli , K. Kepic , et al. 2022. “Effects of Pre‐Term Birth on the Cardio‐Respiratory Responses to Hypoxic Exercise in Children.” Life 12: 79. 35054472 10.3390/life12010079 PMC8777779

[cph470049-bib-0035] Owen‐Jones, Z. , A. Perrochon , E. Hermand , L. Ponthier , L. Fourcade , and B. Borel . 2020. “Evolution of Muscular Oxygenation During a Walking Test in Preterm Children.” Journal of Pediatrics 227: 142–148. 32750391 10.1016/j.jpeds.2020.07.082

[cph470049-bib-0036] Proctor, D. N. , K. C. Beck , P. H. Shen , T. J. Eickhoff , J. R. Halliwill , and M. J. Joyner . 1998. “Influence of Age and Gender on Cardiac Output‐VO_2_ Relationships During Submaximal Cycle Ergometry.” Journal of Applied Physiology (1985) 84: 599–605. 10.1152/jappl.1998.84.2.599 9475871

[cph470049-bib-0037] Raju, T. N. K. , A. S. Buist , C. J. Blaisdell , M. Moxey‐Mims , and S. Saigal . 2017. “Adults Born Preterm: A Review of General Health and System‐Specific Outcomes.” Acta Paediatrica 106: 1409–1437. 28419544 10.1111/apa.13880

[cph470049-bib-0038] Rogers, M. , T. B. Fay , M. F. Whitfield , J. Tomlinson , and R. E. Grunau . 2005. “Aerobic Capacity, Strength, Flexibility, and Activity Level in Unimpaired Extremely Low Birth Weight (<or=800 g) Survivors at 17 Years of Age Compared With Term‐Born Control Subjects.” Pediatrics 116: e58–e65. 15997047 10.1542/peds.2004-1603

[cph470049-bib-0039] Schuermans, A. , and A. J. Lewandowski . 2022. “Understanding the Preterm Human Heart: What Do we Know So Far?” Anatomical Record 305: 2099–2112. 10.1002/ar.24875 PMC9542725 35090100

[cph470049-bib-0040] Sun, X. G. , J. E. Hansen , R. J. Oudiz , and K. Wasserman . 2001. “Exercise Pathophysiology in Patients With Primary Pulmonary Hypertension.” Circulation 104: 429–435. 11468205 10.1161/hc2901.093198

[cph470049-bib-0041] Telles, F. , N. McNamara , S. Nanayakkara , et al. 2020. “Changes in the Preterm Heart From Birth to Young Adulthood: A Meta‐Analysis.” Pediatrics 146: e20200146. 32636236 10.1542/peds.2020-0146

[cph470049-bib-0042] Tetri, L. H. , G. M. Diffee , G. P. Barton , et al. 2018. “Sex‐Specific Skeletal Muscle Fatigability and Decreased Mitochondrial Oxidative Capacity in Adult Rats Exposed to Postnatal Hyperoxia.” Frontiers in Physiology 9: 326. 29651255 10.3389/fphys.2018.00326 PMC5884929

[cph470049-bib-0043] Tikanmaki, M. , N. Kaseva , T. Tammelin , et al. 2017. “Leisure Time Physical Activity in Young Adults Born Preterm.” Journal of Pediatrics 189: 135–142. 28751124 10.1016/j.jpeds.2017.06.068

[cph470049-bib-0044] Vollsaeter, M. , K. Skromme , E. Satrell , et al. 2015. “Children Born Preterm at the Turn of the Millennium Had Better Lung Function Than Children Born Similarly Preterm in the Early 1990s.” PLoS One 10: e0144243. 26641080 10.1371/journal.pone.0144243 PMC4671691

[cph470049-bib-0045] Vrijlandt, E. J. , J. Gerritsen , H. M. Boezen , R. G. Grevink , and E. J. Duiverman . 2006. “Lung Function and Exercise Capacity in Young Adults Born Prematurely.” American Journal of Respiratory and Critical Care Medicine 173: 890–896. 16456146 10.1164/rccm.200507-1140OC

[cph470049-bib-0046] Wagner, P. D. 1988. “An Integrated View of the Determinants of Maximum Oxygen Uptake.” Advances in Experimental Medicine and Biology 227: 245–256. 3381700 10.1007/978-1-4684-5481-9_22

[cph470049-bib-0047] Wagner, P. D. 1991. “Central and Peripheral Aspects of Oxygen Transport and Adaptations With Exercise.” Sports Medicine 11: 133–142. 2047621 10.2165/00007256-199111030-00001

